# Melting and Re-Freezing Leads to Irreversible Changes in the Morphology and Molecular-Level Dynamics of Pfizer-BioNTech COVID-19 Vaccine

**DOI:** 10.3390/medicina57121343

**Published:** 2021-12-09

**Authors:** Eugene Mamontov, Luke L. Daemen, Eric Novak, Matthew B. Stone

**Affiliations:** Oak Ridge National Laboratory, Neutron Scattering Division, Oak Ridge, TN 37831, USA; daemenll@ornl.gov (L.L.D.); novakec@ornl.gov (E.N.); stonemb@ornl.gov (M.B.S.)

**Keywords:** COVID-19, vaccine, thermal stability, quasielastic neutron scattering, inelastic neutron scattering

## Abstract

*Background and Objectives*: As an mRNA-based vaccine, the Pfizer-BioNTech COVID-19 vaccine has stringent cold storage requirements to preserve functionality of the mRNA active ingredient. To this end, lipid components of the vaccine formulation play an important role in stabilizing and protecting the mRNA molecule for long-term storage. The purpose of the current study was to measure molecular-level dynamics as a function of temperature in the Pfizer-BioNTech COVID-19 vaccine to gain microscopic insight into its thermal stability. *Materials and Methods*: We used quasielastic and inelastic neutron scattering to probe (1) the vaccine extracted from the manufacturer-supplied vials and (2) unperturbed vaccine in the original manufacturer-supplied vials. The latter measurement was possible due to the high penetrative power of neutrons. *Results*: Upon warming from the low-temperature frozen state, the vaccine in its original form exhibits two-step melting, indicative of a two-phase morphology. Once the melting is completed (above 0 °C), vaccine re-freezing cannot restore its original two-phase state. This observation is corroborated by the changes in the molecular vibrational spectra. The molecular-level mobility measured in the resulting single-phase state of the re-frozen vaccine greatly exceeds the mobility measured in the original vaccine. *Conclusions*: Even a brief melting (above 0 °C) leads to an irreversible alteration of the two-phase morphology of the original vaccine formulation. Re-freezing of the vaccine results in a one-phase morphology with much increased molecular-level mobility compared to that in the original vaccine, suggesting irreversible deterioration of the vaccine’s in-storage stability. Neutron scattering can be used to distinguish between the vibrational spectra characteristic of the original and deteriorated vaccines contained in the unperturbed original manufacturer-supplied vials.

## 1. Introduction

Prior to the COVID-19 pandemic, development and production of a new vaccine required many years. However, Pfizer-BioNTech and Moderna vaccines against SARS-CoV-2 were developed and mass-produced in an unprecedented short amount of time. This was possible, in large part, because they are RNA vaccines. When the RNA of such a vaccine is introduced into a host, it acts as a messenger RNA (mRNA) to induce production of the intended foreign protein by the host, thereby stimulating an adaptive immune response and building up immunity to the pathogen, of which the intended foreign protein is a part. The prime benefit of using mRNA to make the host cells produce the antigen is that mRNA is much easier to manufacture than antigen proteins or attenuated virus, as commonly used in traditional vaccines. This greatly expedites the design and production of RNA vaccines [1]. Furthermore, since RNA vaccines are not made of a pathogen (either activated, or inactivated), they are not infectious. It is widely believed that, following the pioneering and widespread use of RNA technology in COVID-19 vaccines, many future vaccines for transmittable diseases, as well as cancer immunotherapy, will use RNA technology.

Despite its many advantages, RNA vaccines rely on a comparatively fragile mRNA molecule that degrades within minutes in an exposed environment or even faster in an organism. Thus, mRNA molecules must be encapsulated in a protective coating, for both storage and in vivo delivery. Lipid nanoparticles (LNPs) have emerged as the most promising delivery vehicle for exogenous mRNA [2]. LNPs are complex nano-structured entities protecting the encapsulated RNA molecule, whose molecular architecture is critical for the storage/delivery function of the vaccine. For example, Pfizer-BioNTech vaccine must be stored at −80 to −60 °C, whereas Moderna vaccine can be stored at −25 to −15 °C. It is believed that differences in LNPs formulations could account for the thermostability differences among various vaccines, at present and in the future.

The publicly disclosed [3] chemical composition of the Pfizer-BioNTech vaccine is as follows: “Each 0.3 mL dose of the Pfizer-BioNTech COVID-19 Vaccine contains 30 mcg of a nucleoside-modified messenger RNA (modRNA) encoding the viral spike (S) glycoprotein of SARS-CoV-2. Each dose of the Pfizer-BioNTech COVID-19 Vaccine also includes the following ingredients: lipids (0.43 mg (4-hydroxybutyl)azanediyl)bis(hexane-6,1-diyl)bis(2-hexyldecanoate), 0.05 mg 2[(polyethylene glycol)-2000]-N,N-ditetradecylacetamide, 0.09 mg 1,2-distearoyl-sn-glycero-3-phosphocholine, and 0.2 mg cholesterol), 0.01 mg potassium chloride, 0.01 mg monobasic potassium phosphate, 0.36 mg sodium chloride, 0.07 mg dibasic sodium phosphate dihydrate, and 6 mg sucrose.” In addition, it is stated [3] that “The Pfizer-BioNTech COVID-19 Vaccine is supplied as a frozen suspension in multiple dose vials; each vial must be diluted with 1.8 mL of sterile 0.9% Sodium Chloride Injection, USP prior to use to form the vaccine. After dilution, each vial contains 5 doses of 0.3 mL per dose. The Pfizer-BioNTech COVID-19 Vaccine does not contain a preservative.” As the vaccine is not a lyophilized powder, but a frozen suspension, it may also contain unspecified amounts of aqueous media.

Unlike the overall chemical composition, the information on the exact vaccine’s structural organization may be proprietary (see [4] for a review of general structural components of the mRNA-based vaccines). However, even without the information on the exact vaccine’s structure, the molecular-level dynamics of the vaccine’s constituents could be efficiently probed by quasielastic/inelastic neutron scattering (QENS/INS). Incoherent QENS/INS, which probes H-weighed scattering signal, will predominantly measure composition-weighed scattering from inactive (other than RNA) ingredients in the vaccine formulation that dominate the vaccine’s chemical composition. Furthermore, neutron scattering techniques can advantageously use high penetration power of neutrons and their high sensitivity to H to measure the samples stored in original unopened manufacturer-supplied glass vials, thereby closely mimicking the vaccine’s storage conditions.

While our earlier QENS/INS studies (see [5] and references therein) were aimed at elucidating the molecular-level dynamics in potential COVID-19 drugs, the main aim of the present work is to investigate the influence of the storage thermal history on the molecular-level dynamics in Pfizer-BioNTech COVID-19 vaccine. We have found that even a brief melting (above 0 °C) leads to an irreversible alteration of the two-phase morphology of the original vaccine formulation. Re-freezing of the vaccine results in a one-phase morphology with much increased molecular-level mobility of the constituents compared to that in the original vaccine, suggesting irreversible deterioration of the vaccine’s in-storage stability after even a brief melting event.

## 2. Materials and Methods

The neutron scattering experiments were performed using the QENS spectrometer BASIS [6] and the INS spectrometer VISION [7] at the Spallation Neutron Source, Oak Ridge National Laboratory, USA. For BASIS, the scattering signal was integrated over the scattering momentum transfer range of 0.2 Å^−1^ < Q < 1.2 Å^−1^ and normalized to a vanadium standard. For VISION, the background spectrum (from an empty vial in an aluminum cell) was measured separately and subtracted from the vaccine’s scattering signal. The energy resolution at the elastic line was ca. 0.0037 meV and 0.12 meV for BASIS and VISION, respectively. The usable range of energy transfers was ±0.1 meV for BASIS and up to 550 meV for VISION. The temperatures and duration of the measurements are discussed in detail in the Results section. 

The raw data collected at BASIS and VISION were reduced using the routines implemented in the standard package for data reduction utilized at SNS [8]. QENS data fitting was carried out using the DAVE software package [9]. Both VISION and BASIS measure the intensity, I(Q, E), of neutrons scattered by the sample as a function of energy transfer, E, between the neutrons and the sample, similar to, e.g., Raman spectroscopy (the conversion factor is approximately 1 meV = 8 cm^−1^). On both spectrometers, the neutrons scattered with an energy transfer much smaller than the spectrometer’s energy resolution (elastic line width) are perceived as scattered elastically (indistinguishable from the truly elastically scattered neutrons with E = 0). VISION is a high-intensity general-purpose vibrational spectrometer optimized for quick (oftentimes, in a few minutes) data collection over a broad range of energy transfers. BASIS is a dedicated high energy-resolution spectrometer, with the elastic line width defined more precisely by a factor of ca. 30 compared to VISION, but much longer data collection (oftentimes, hours for each set of experimental conditions) and a limited energy transfer range. The high energy resolution of BASIS makes it suitable for measuring specifically QENS (as opposed to general INS) signal, which is visible at low energy transfers near the elastic line. 

Manufacturer-supplied vials of Pfizer-BioNTech COVID-19 vaccine were obtained from Oak Ridge National Laboratory’s Health Services Division, where they have been stored at −80 to −60 °C. The vaccine samples were loaded into sample holders for neutron scattering experiments the day after the vaccine expiration date. Thus, while rendered unsuitable for administering in humans, the vaccine was unlikely to differ substantially from its state immediately before the expiration date. This is because possible in-storage degradation of the vaccine would be gradual, and an extended cold storage time that is short compared to the 180 days storage limit as specified by the manufacturer should not lead to sudden substantial changes in the state of the vaccine.

All measured samples were designated as either “in original vial” or “out of original vial”. The former samples were loaded by placing an original unopened vial with vaccine into a 19.05 mm inner diameter cylindrical, indium wire-sealable, aluminum sample holder. A small sheath handmade of a flexible neutron-absorbing cadmium sheet was placed over the top of the vial to prevent exposure of the rubber plug to the neutron beam. This was done to avoid potential contamination of the measured signal by scattering from the H-rich rubber material. In addition, the vaccine label was carefully scraped off the vial using a scalpel and the glue underneath the label was removed using ethanol. This was also done to prevent contamination of the measured signal by scattering from the H-rich paper and glue materials. All these manipulations were carried out while the vial was kept in a dry ice box or submerged in liquid nitrogen, and the indium-sealed aluminum sample holder with the enclosed original vial with the vaccine was then stored in the dry ice box before the neutron scattering experiments commenced. Unlike the rubber, paper, and glue materials, the aluminum sample holder and glass vial give rise only to the relatively weak neutron scattering signal, thus allowing collection of the scattering signal dominated by the H-rich vaccine in the vial. The “out of original vial” sample for BASIS was loaded using a metal spatula in indium wire-sealable flat-plate geometry aluminum sample holders, 50 mm tall, 30 mm wide, and 0.5 mm thick. Thus, the scattering geometry of the samples “out of original vial” was greatly different from the geometry of the samples “in original vial”, as the latter resided primarily on the bottom of the glass vial. The higher neutron beam-exposed sample area is beneficial for increasing the signal statistics due to intercepting more of the incident neutron beam height. While the full neutron beam height of 30 mm was used at BASIS for the sample “out of original vial”, the beam height was reduced to 20 mm for the samples “in original vial”. At the same time, the smaller thickness of the samples in the flat-plate sample holders is beneficial for suppressing the effects of multiple neutron scattering in the samples. That is, the samples “out of original vial” have much more favorable scattering geometry, whereas the samples “in original vial” exactly represent the storage conditions of the vaccine (such as contact with the glass as opposed to aluminum surface, etc.) 

## 3. Results

### 3.1. QENS Measurements

**Figure 1:**
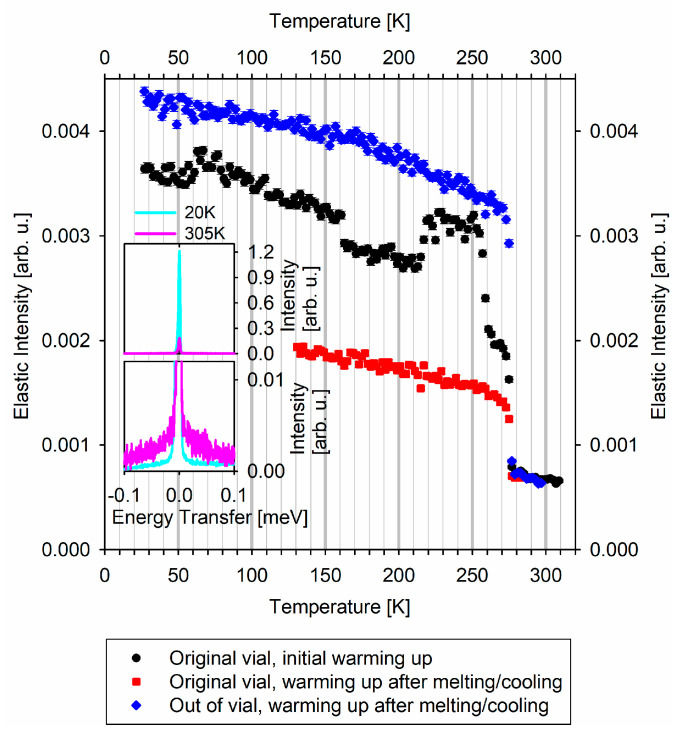
QENS spectra-derived “elastic” scattering intensities measured at BASIS neutron spectrometer. Top inset: QENS spectra from the sample in the original vial (on the initial warming up) measured at 20 K and 305 K. Bottom inset: the same QENS spectra as in the top inset with y-axis truncated to emphasize the QENS signal at 305 K.

The first QENS measurement was from the sample “in original vial”. The sample was mounted, directly from the dry ice box, on a stick, which was quickly immersed into a closed-cycle refrigerator (CCR) pre-cooled to T = 200 K that was installed at BASIS. The CCR with the sample was then cooled down to the baseline temperature of 20 K, which took about 2 h. Then a QENS spectrum was collected at T = 20 K for 8 h (cyan line in Figure 1 insets). After that, a temperature scan of the “elastic” scattering intensity (the intensity integrated over −0.0037 to +0.0037 meV range of the QENS spectra) commenced at a heating rate of 5 K per hour. The data recorded at 2 K intervals (about every 24 min) are presented with black symbols in the main panel of Figure 1. Since full QENS spectra are collected at BASIS by default, each point of the “elastic” intensity temperature scan is obtained by the integration of the QENS intensity over −0.0037 to +0.0037 meV, that is, over ±HWHM (half-width at half maximum) of the energy resolution line. The pink line in the Figure 1 insets shows the full QENS spectrum summed up for 300 K < T < 310 K (average T = 305 K), that is, the QENS spectra collected for 2 h (compared to 8 h for the T = 20 K spectrum in Figure 1 insets). The total collection time for the black symbols in the main panel of Figure 1 was about 57 h.

After that, the sample was cooled down to T = 20 K in about 4 h, and a QENS spectrum was collected at T = 20 K for 4 h. Then the sample was quickly warmed up to T = 130 K, and another temperature scan of the “elastic” scattering intensity commenced at a heating rate of 5 K per hour. This data set recorded at 2 K intervals (about every 24 min) is presented with red symbols in the main panel of Figure 1. The total collection time for the red symbols was about 36 h.

The second QENS measurement was from the sample “out of original vial”. The sample was mounted on a stick, which was quickly immersed into a CCR pre-cooled to T = 200 K that was installed at BASIS. The CCR with the sample was then cooled down to the baseline temperature of 20 K, which took about 2 h. Then a QENS spectrum was collected at T = 20 K for 4 h. After that, analogously to the measurement of the sample “in original vial”, a temperature scan of the “elastic” scattering intensity commenced at a heating rate of 5 K per hour. The data recorded at 2 K intervals (about every 24 min) are presented with blue symbols in the main panel of Figure 1. The total collection time for the blue symbols in the main panel of Figure 1 was about 57 h.

**Figure 2:**
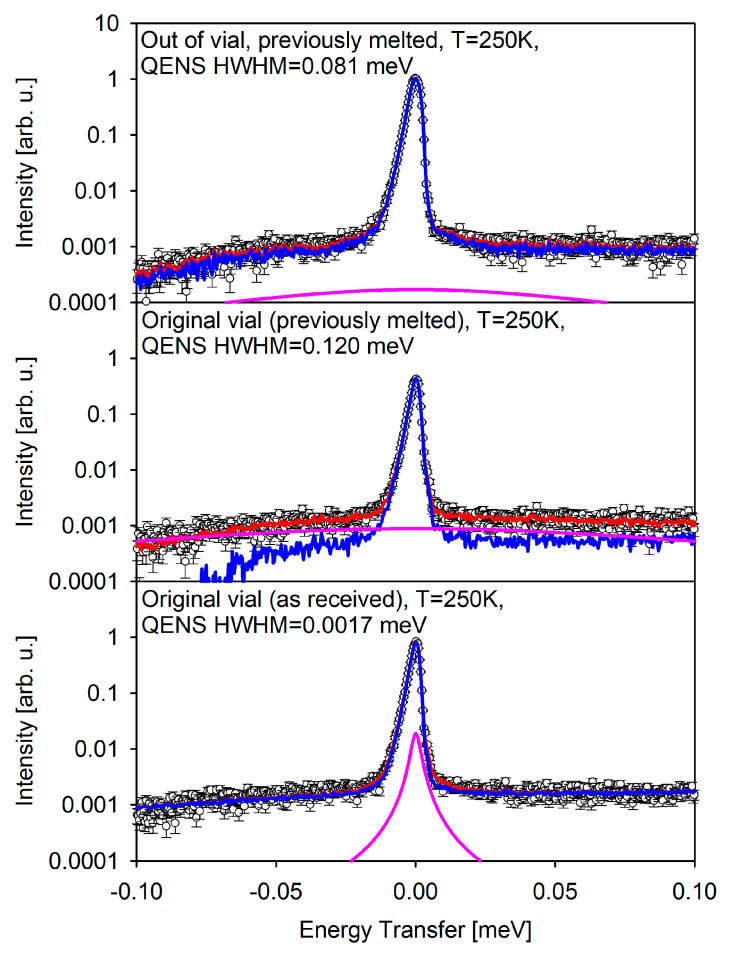
QENS spectra measured at BASIS neutron spectrometer (symbols) presented as intensity in arbitrary units as a function of energy transfer in meV. Red line: overall fit to the data. Blue line: elastic component of the overall fit plus background. Pink line: quasielastic component of the overall fit.

Bottom, middle, and top panels of Figure 2 show the fitting analysis of the QENS spectra associated, respectively, with the black, red, and blue symbols temperature scan measurements presented in Figure 1. The full QENS spectrum summed up for 245 K < T < 255 K (average T = 250 K), that is, the spectra collected for 2 h, are presented by the open symbols in Figure 2.

The data were fitted to the following model:

I(E) = [xδ(E) + (1 − x)S(E)] ⊗ R(E) + B(E),(1)

where a model function in the form of a Lorentzian, S(E) = HWHM/(π(E^2^ + HWHM^2^)), describing the intrinsic molecular-level dynamics in the sample, is superimposed with a delta-function, δ(E), which accounts for the elastic signal with a spectral weight of 0 < x < 1, and together they are convolved with the resolution function, R(E). Each of the three T = 250 K data sets presented in Figure 2 has its own resolution function, measured at T = 20 K as explained above. The B(E) is a linear background term, B(E) = (C_1_ + C_2_E).

### 3.2. INS Measurements

Unlike a QENS signal that originates from stochastic molecular motions and thus needs to be measured at finite temperatures, a vibrational INS signal is best measured at the baseline temperature; in the current experiment, at T = 5 K. 

**Figure 3:**
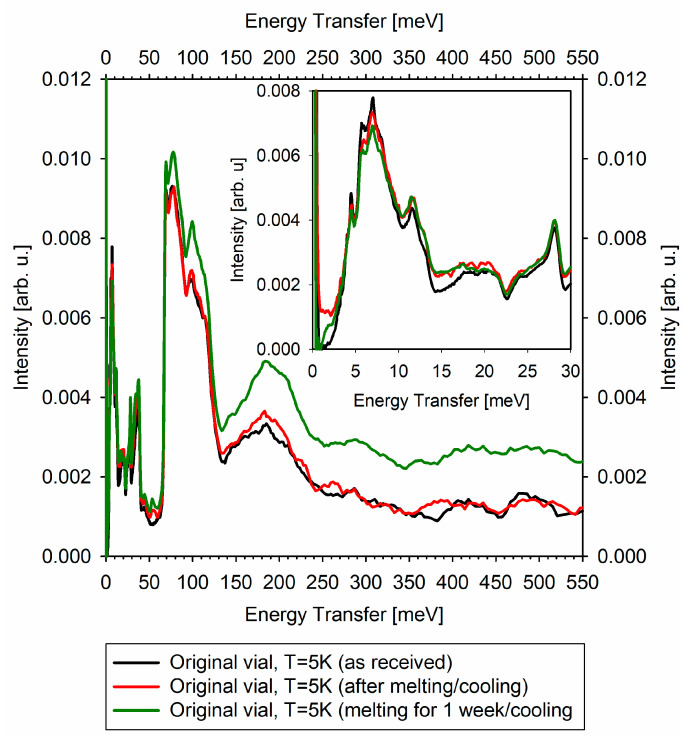
INS spectra measured at the VISION neutron spectrometer (the elastic peak maxima are normalized to unity). The inset shows zoomed-in spectra to emphasize the difference in the signal at low energy transfer, particularly, 0–3 meV.

Comparison of the never melted and melted/re-frozen vaccines (one re-frozen immediately after melting, the other re-frozen after spending a week in the melted state) is presented in Figure 3. The difference in the vaccines’ vibrational spectra is especially pronounced in the region of the very soft vibrational modes, at 0–3 meV. In addition, there may be a new mode developing in the melted vaccines at 55 meV. The spectral features in the 350–550 meV range tend to become less defined from the never-melted to briefly-melted to long-time-melted vaccine. There seems to be relative increase in the intensity of the inelastic features in the long-melted vaccine. Importantly, each of the high-quality vibrational spectra presented in Figure 3 was collected in just under 2 h.

## 4. Discussion

The evolution of the “elastic” scattering intensity as a function of temperature is illustrated in the insets of Figure 1. A sharp drop in the measured “elastic” scattering intensity upon heating is associated with a phase transition such as melting. This behavior of the data occurs when the wings-like QENS signal originating from the molecular mobility in the liquid state grows at the expense of the sharp elastic line. In the absence of phase transitions, the “elastic” scattering intensity tends to decrease gradually when the temperature is raised, similar to the decrease in Bragg peak intensity in diffraction data at high temperatures (Debye–Waller thermal factor). While abrupt drops in the “elastic” scattering intensity due to melting are commonly observed, intensity increases with the increasing temperature are uncommon, yet have been observed in the cases of crystallization on warming up from the low-temperature amorphous state that may precede melting [10]. Thus, such increases for the sample “in original vial”, presented with black symbols in the main panel of Figure 1, could possibly be associated with crystallization on warming up. Interestingly, this increase occurs around 215 K (−58 °C), which is the upper limit for the storage requirements (−60 °C). At the same time, the sharp drops in the “elastic” scattering intensity at 260 and 273 K must be unequivocally attributed to melting of the vaccine “in original vial”. The melting point of 273 K indicates melting of the bulk-like aqueous phase, whereas the melting point of 260 K is due to melting of another phase. This could represent either a lipid phase melting, or melting of an aqueous-based phase, with ca. 13 K melting point depression. This melting point depression in an aqueous phase may arise from either the effect of water confinement (e.g., in vesicles) or solutes in water (e.g., sucrose), or as a cumulative confinement/solute effect. In any case, the melting of the vaccine “in original vial” demonstrates that this vaccine has two-phase morphology, with one of the phases being bulk-like water.

Once the vaccine “in original vial” has been re-frozen following the melting, another heating cycle, as represented by the red symbols in the main panel of Figure 1, shows that the two-phase vaccine morphology that gave rise to the two-step melting in the first heating cycle could not be restored by re-freezing. The melted and re-frozen vaccine exhibits a single “elastic” intensity drop on warming up, lacking the second phase melting step at 260 K and the preceding “elastic” intensity increase during crystallization. The second phase, which gave rise to the melting step at 260 K in the original, never melted, vaccine, disappears irreversibly upon the vaccine melting, leaving behind the bulk-like aqueous-based phase that melts at 273 K, showing no melting point depression.

To verify independently the irreversible change in the vaccine morphology upon melting, we probed the sample “out of original vial”, which showed visible melting while being loaded in the aluminum sample holder. The “elastic” scattering intensity scan for this sample of pre-melted vaccine (blue symbols in the main panel of Figure 1) shows a single “elastic” intensity drop at 273 K upon warming, indicative of the presence of the single bulk-like aqueous-based phase in the re-frozen vaccine sample “out of original vial”.

Having found a morphological feature in the original vaccine formulation that seems to disappear irreversibly upon melting and re-freezing, we attempt to analyze the difference in the molecular-level dynamics between the never melted and melted/re-frozen vaccine. To this end, we analyzed the full QENS spectra averaged for 245 K < T < 255 K (average T = 250 K) that were used to obtain the “elastic” scattering intensity points of black, red, and blue curves in the main panel of Figure 1. This temperature region was chosen to encompass the maximum temperatures (but still below the onset of the melting process) to ensure that the stochastic motions could be measurable with the BASIS energy resolution. The fit component of the principal interest to us, the (1 − x)S(E) ⊗ R(E) term in Equation (1), is presented by the pink line in Figure 2 for the three measurements. In the never melted sample “in original vial”, the HWHM of the S(E) term describing the QENS signal is 0.0017 meV, which corresponds to the characteristic relaxation time of ca. 390 ps. On the other hand, in the melted/re-frozen samples, the QENS signal’s HWHM becomes 0.120 meV (corresponding to ca. 6 ps) and 0.081 meV (corresponding to ca. 8 ps) for the samples “in original vial” and “out of original vial”, respectively. The 50% discrepancy is likely due to multiple scattering in the melted/re-frozen sample “in original vial”, as scattering from thick samples is known to broaden the measured QENS signal beyond its intrinsic width [11,12]. It should be noted that the as received frozen vaccine, which is visibly distributed between the bottom and the walls of the glass vial in the never melted sample “in original vial”, tends to flow entirely to the bottom of the vial in the course of melting. This results in the effectively higher sample thickness and more pronounced self-shielding and multiple scattering in the melted/re-frozen sample. This observation is corroborated by the decreased intensity of the red symbols curve vs. the black symbols curve in the main panel of Figure 1, due to the fact that the self-shielding has significantly increased in the melted/re-frozen sample “in original vial”.

There is a dramatic difference, by at least a factor of 50, between the never melted and melted/re-frozen state of the vaccine in the characteristic relaxation time measured by QENS at T = 250 K. The compositional complexity of the vaccine precludes assignment of the measured relaxation time to a specific chemical component. However, it is evident that the melted/re-frozen vaccine state is characterized by the much more pronounced molecular-level dynamics, which is indicative of the molecular-level “softening” and is expected to correlate inversely with the cold storage stability of the vaccine. Importantly, INS measurements, as presented in Figure 3, show the development of the very soft (in the 0–3 meV region) vibrational modes in the melted/re-frozen vaccines, corroborating the QENS observations.

The apparent relative increase in the intensity of the inelastic features in the long-melted vaccine compared to the briefly melted vaccine in Figure 3 illustrates the evolution of the vaccine state after melting. Because the vaccine needs to be melted before dilution and administration, and, moreover, can be stored for a certain period of time in the melted state, it is evident that the irreversible changes to the vaccine morphology upon melting do not lead to the quick degradation of the active mRNA component. However, our results suggest that even a prompt re-freezing of the vaccine after a melting event could not be used to extend the vaccine’s cold storage time since even the promptly re-frozen vaccine already exhibits increased “softness” at the molecular level, likely adverse to the in-storage stability. This is because the molecular-level processes of degradation (regardless of their mechanism) may proceed at a faster rate in the re-frozen vaccine characterized by the increased molecular-level mobility. It should be noted that the current study cannot analyze the vaccine’s in-storage stability in relation to the evolution of its efficacy.

The crystallization that we observe in the original vaccine upon warming it up above 215 K is a change that would not be reversed by returning from T < 260 K to T = 200 K. Even though, as mentioned above, T = 215 K (−58 °C) is very close to the manufacturer-specified upper limit for the storage requirements (−60 °C), the current study does not suggest a mechanism for degradation of the vaccine in the crystallized state as long as it remains below the first melting point at 260 K.

Pfizer and BioNTech announced development of lyophilized formulation of the vaccine for improved storage and thermal stability [13]. QENS/INS can be expected to play an important role in characterization of the freeze-dried vaccine formulations. Compared to other microscopical molecular-level and macroscopical phase-level dynamic characterization techniques that can yield comparable information on lyophilized pharmaceuticals, QENS considered alone may not possess the specificity of NMR spectroscopy [14,15] and the quickness and easiness of use of differential scanning calorimetry [15,16]; however, QENS ability to probe the molecular-level and phase-level dynamics directly in manufacturer-supplied packaging, which is due to the high penetrative power of neutrons, is unparalleled. In addition, a combination of QENS and INS can greatly alleviate the inconvenience of time-consuming measurements associated with the former technique. For example, in the present study, the QENS experiment, carried out over several days, was used to demonstrate the irreversible change in the original vaccine induced by melting and show that the re-frozen vaccine becomes more dynamic (that is, “softer”) at the molecular level. This relatively time-consuming observation has been linked to the emergence of the “soft” vibrational modes in the melted and re-frozen vaccine, which can be verified in a quick INS measurement, still on the vaccines contained in the original manufacturer-supplied vials. That is, a one-time, even though somewhat time-consuming, QENS measurement helped establish how a quick INS measurement can be used for a fast and non-destructive check of whether a vaccine in the seemingly intact vial has undergone melting, which could be detrimental for the long-term storage stability of the vaccine. This could be especially helpful for a vaccine that might have been briefly exposed to warmer temperatures. A quick excursion above the melting temperature might be difficult to emulate for studies using relatively slow QENS measurements, but, as long as the melting has taken place, the resulting changes will readily manifest themselves in the INS measurement of the final state of the vaccine.

## 5. Conclusions

Upon warming from the low-temperature frozen state, the Pfizer-BioNTech vaccine in the original unopened vial showed two-step melting, indicative of a two-phase morphology. The melting point of 273 K indicated melting of bulk-like aqueous phase, whereas the melting point of 260 K was due to melting of another phase. Once the melting was completed (above 0 °C), the re-frozen vaccine lost its original two-phase state, exhibiting a single melting step at 273 K. Quasielastic neutron scattering measurements at 250 K showed about 50 times faster molecular-level dynamics in the melted and re-frozen vaccine compared to the original vaccine, which is likely detrimental to the long-term cold storage stability of the re-frozen vaccine. Concurrently, inelastic neutron scattering measurements showed development of “soft” vibrational modes in the re-frozen vaccine in the 0–3 meV range. We concluded that even a brief melting (above 0 °C) leads to an irreversible alteration of the two-phase morphology of the original vaccine. Re-freezing of the vaccine results in a one-phase morphology with much increased molecular-level mobility and “softness”, suggesting irreversible deterioration of the vaccine’s cold-storage stability.

## Data Availability

The data collected in the neutron scattering experiments are available upon request to the authors.
